# Human Adipose Cells *In Vitro* Are Either Refractory or Responsive to Insulin, Reflecting Host Metabolic State

**DOI:** 10.1371/journal.pone.0119291

**Published:** 2015-03-13

**Authors:** Vladimir A. Lizunov, Karin G. Stenkula, Paul S. Blank, Aaron Troy, Jo-Ping Lee, Monica C. Skarulis, Samuel W. Cushman, Joshua Zimmerberg

**Affiliations:** 1 Program in Physical Biology, Eunice Kennedy Shriver National Institute of Child Health and Human Development, National Institutes of Health, Bethesda, MD, United States of America; 2 Experimental Diabetes, Metabolism, and Nutrition Section, National Institute of Diabetes and Digestive and Kidney Diseases, National Institutes of Health, Bethesda, MD, United States of America; 3 Diabetes, Endocrinology, and Obesity Branch, National Institute of Diabetes and Digestive and Kidney Diseases, National Institutes of Health, Bethesda, MD, United States of America; 4 Experimental Medical Sciences, Lund University, Lund, Sweden; Boston University School of Medicine, UNITED STATES

## Abstract

While intercellular communication processes are frequently characterized by switch-like transitions, the endocrine system, including the adipose tissue response to insulin, has been characterized by graded responses. Yet here individual cells from adipose tissue biopsies are best described by a switch-like transition between the basal and insulin-stimulated states for the trafficking of the glucose transporter GLUT4. Two statistically-defined populations best describe the observed cellular heterogeneity, representing the fractions of refractive and responsive adipose cells. Furthermore, subjects exhibiting high systemic insulin sensitivity indices (SI) have high fractions of *responsive* adipose cells *in vitro*, while subjects exhibiting decreasing SI have increasing fractions of *refractory* cells *in vitro*. Thus, a two-component model best describes the relationship between cellular refractory fraction and subject SI. Since isolated cells exhibit these different response characteristics in the presence of constant culture conditions and milieu, we suggest that a physiological switching mechanism at the adipose cellular level ultimately drives systemic SI.

## Introduction

Adipose tissue plays a key role in lipid storage, glucose homeostasis, and the regulation of energy metabolism; defects in adipose cell function are associated with the development of insulin resistance and type 2 diabetes [1–4]. The use of high resolution microscopy methods, such as total internal reflection fluorescence and super-resolution localization, on isolated adipose cells allows us to observe and analyze the fundamental dynamic trafficking events that comprise a response to insulin at the level of individual secretory vesicles and proteins [5, 6]. Systemic insulin responses, such as glucose clearance, represent the integrated GLUT4 translocations of all responding muscle and adipose cells. Genetic, epigenetic, dietary, and lifestyle factors (including exercise) clearly influence population-level adipose cell insulin responsiveness [4, 7, 8]. On the other hand, as we have recently shown directly at the GLUT4 trafficking level, populations of isolated human adipose cells in *in vitro* culture media lacking host serum retain the wide variation in insulin sensitivity of their hosts [4, 9]. However, the relationship between adipose cell heterogeneity and development of insulin resistance may be masked when population responses are measured. Is insulin resistance due to a graded loss of insulin response at the individual cellular level or does it reflect changes in the fraction of cells responding to insulin? Sporadic reports have described unusually high degrees of heterogeneity in the insulin response of single adipose cells, starting with the pioneering work of Gliemann [10]. A fat-specific insulin receptor knockout in mice gave similar results [11], as did individual 3T3-l1 adipocytes [12]. Here, we analyze the relationship between human adipose cell heterogeneity and subject systemic insulin resistance by taking advantage of the GLUT4 trafficking response data we previously reported as average population values for the adipose cells from each subject [9].

## Results and Discussion

To investigate the link between insulin response heterogeneity at the cellular level and systemic insulin resistance *in vivo*, we analyzed GLUT4 trafficking data in individual human adipose cells isolated from subjects exhibiting a wide range of systemic insulin sensitivity (SI) [9]. We used previously published assays [5] to characterize the adipose cell insulin response by measuring the insulin-induced reduction of GLUT4 storage vesicle (GSV) trafficking along microtubules (referred to as mobility rate) and the subsequent increase in GSV fusion with the plasma membrane (fusion rate).

**Fig 1:**
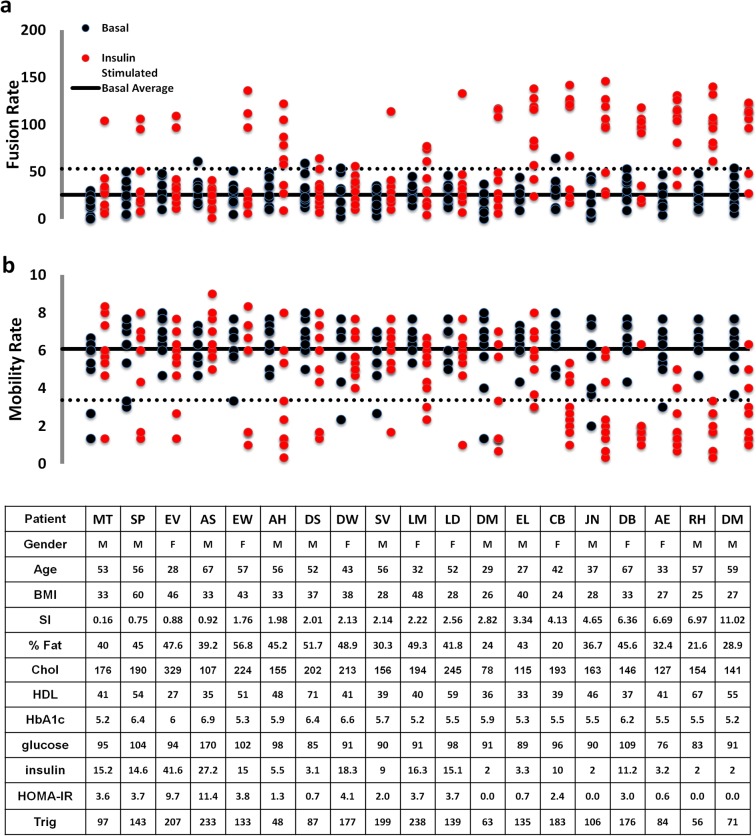
Dot plot of basal and insulin-treated cellular activities, GLUT4 storage vesicle (GSV) fusion and mobility rates, in adipose cells isolated from subjects with varying degrees of insulin sensitivity. For each subject (N = 19), eight cells were evaluated in the basal (black) and insulin-stimulated states (red). Subject ID, gender, age, BMI, SI and %Fat is shown below each group of cell measurements. Both GSV mobility and fusion rates were acquired from time-lapse TIRF recordings of isolated adipose cells transfected with GLUT4-mCherry and IRAP-pHlourin The upper panel (a) shows fusion rates measured in individual cells isolated from each subject. GSV fusion events were detected as spikes of IRAP-pHluorin fluorescence representing expansion of the fusion pore and pH equilibration within the lumen of the fusing GSV. Fusion rate for each cell was calculated as the number of IRAP-pHluorin spikes detected per unit area during one minute of recording. The lower panel (b) shows GSV mobility rates measured as the number of mobile GLUT4-mCherry vesicles detected in the TIRF-zone (within ∼200 μm of the cell membrane). GSV mobility rate was measured in isolated basal or insulin-treated cells and quantified as the number of mobile vesicles (with trajectory length >2 μm) detected within a 10x10 μm region of interest during one minute of recording (60 frames). The solid black lines represent average basal rates calculated from the pooled population of basal cells for each subject. The dotted lines represent 95% confidence intervals for pooled basal values. With increasing SI (from left to right), an increasing number of insulin-stimulated cells exhibit GSV mobility and fusion rates outside the 95% confidence interval calculated from the basal values.

**Fig 2:**
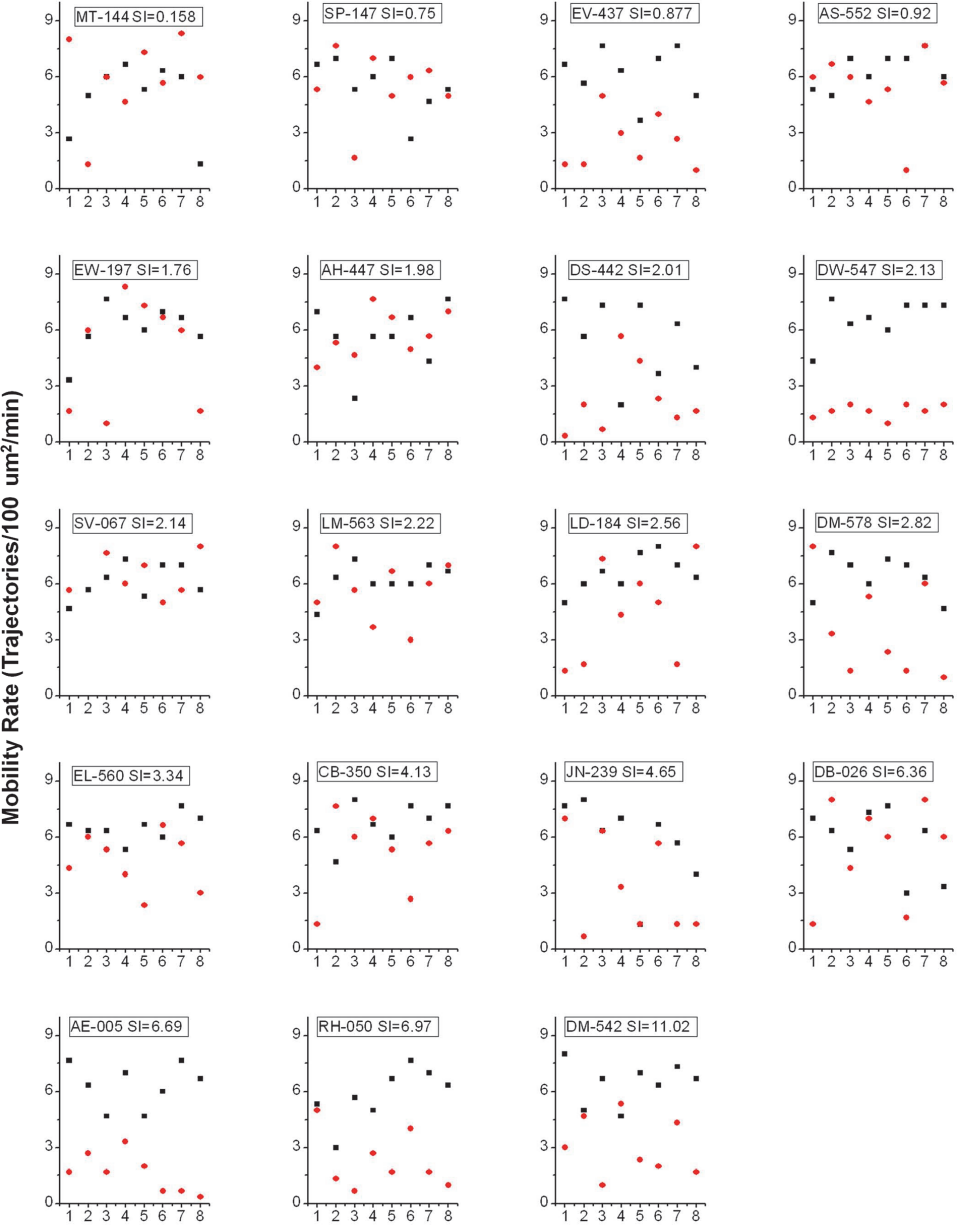
Mobility rate in the basal (black) and insulin-treated (red) states measured in individual cell and plotted for each subject. X axis corresponds to the sequential order in which cells were imaged for each subject. Y axis represents GSV fusion rate measured as the number mobile vesicles per 100 μm²/min.

**Fig 3:**
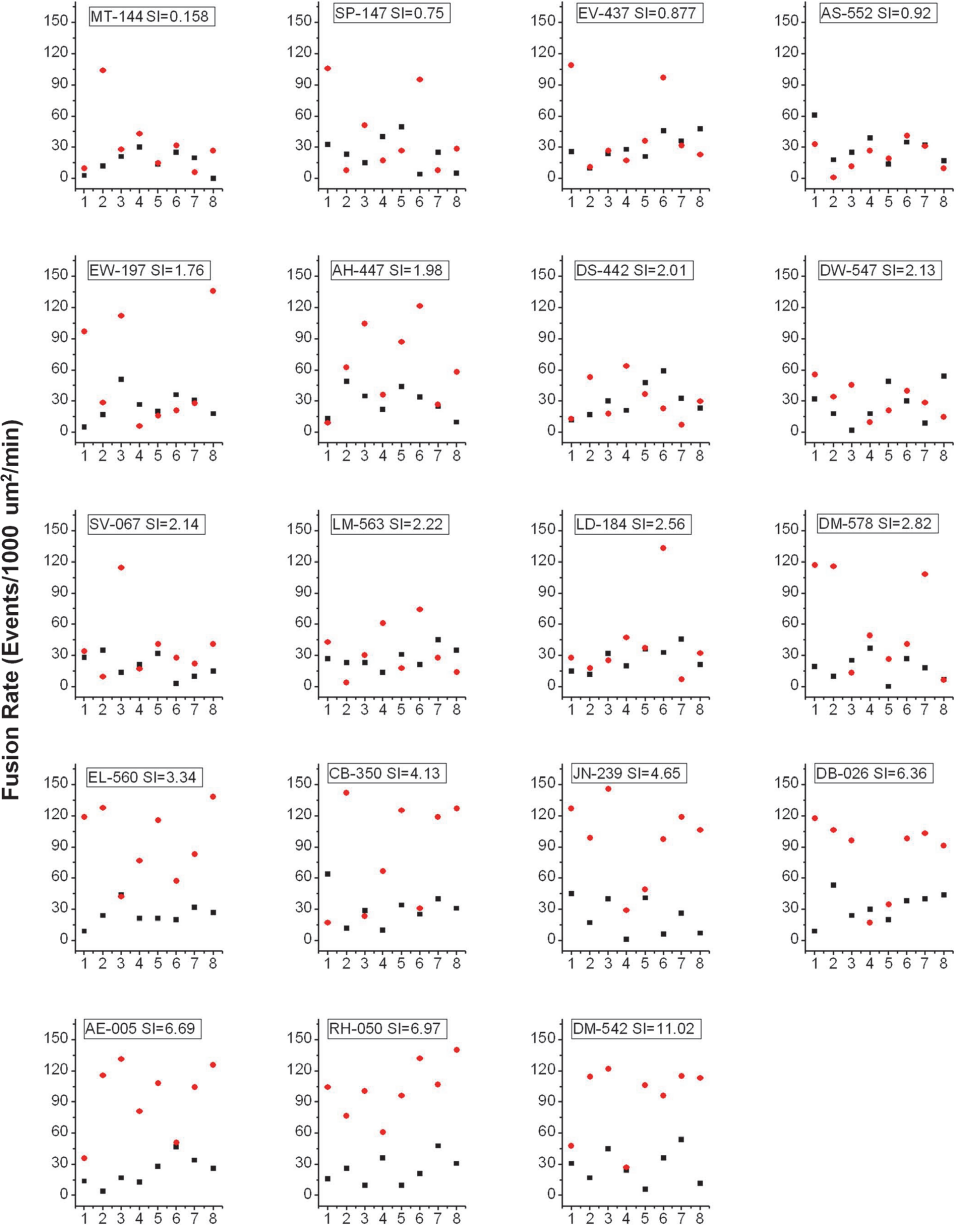
Fusion rate in the basal (black) and insulin-treated (red) states measured in individual cell and plotted for each subject. X axis corresponds to the sequential order in which cells were imaged for each subject. Y axis represents GSV fusion rate measured as the number fusion events detected with IRAP-pHluorin per 1000 μm²/min.

To analyze individual adipose cell responses to insulin, we have utilized statistical methods that avoid data averaging and allow us to identify underlying distributions of cellular responses per subject and among the pooled data from a group of 19 subjects with SI ranging from 0.16 to 11. The data presented in Fig. 1 show GSV mobility and fusion rates measured in individual adipose cells in the basal state (black) and in response to 0.1 IU/ml insulin (maximal stimulation, red), plotted for each subject, with SI increasing from left to right. In order to show all the individual cell responses without masking or overlapping of the individual data points, we also plotted these parameters for each subject in a separate graph (Figs. 2 and 3). Interestingly, the major difference between cells from insulin-resistant subjects (low SI<2) and insulin-sensitive subjects (SI>4) is not the individual cell response amplitude, but rather the *number* of cells that exhibit a 3–4 fold response. Simultaneously, in almost every subject, we observed cells that do not exhibit any insulin response that could be statistically distinguished from the typical basal range of values for mobility and fusion rates (Fig. 1, symbols between the solid black lines representing the average basal rate and the dotted lines representing the 95% confidence intervals). This observed heterogeneity in the insulin response of individual adipose cells strongly indicates that the underlying distribution is far from normal and thus that simple averaging of the cellular data is not appropriate.

### Human adipose cells segregate into two populations: insulin-refractory and insulin-responsive

**Fig 4:**
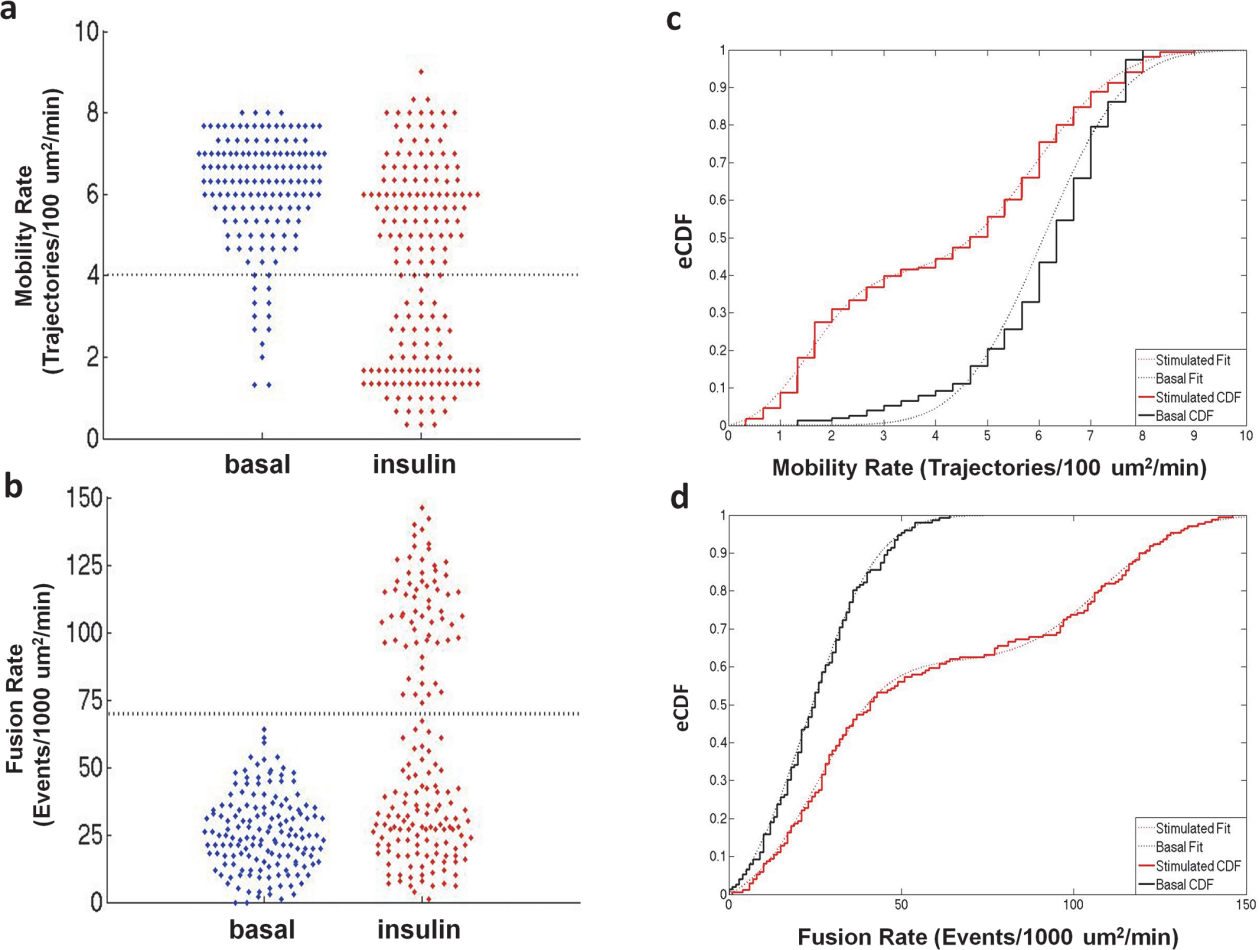
“Bee swarm” plots of single cell GSV mobility (a) and fusion (b) rates measured in the basal and insulin-stimulated states are consistent with two populations in the insulin-stimulated state in which one population matches the basal state. The “bee swarm” plots were created using the plotSpread function (by Jonas) and the cluster boundaries were calculated using the K-means clustering function, both within the program MATLAB. Cumulative distributions calculated for GSV mobility (c) and fusion (d) rates pooled from basal (black) and insulin stimulated (red) cells from all subjects. Basal CDFs were described by a single zero-truncated Gaussian cumulative distribution, while both CDFs for insulin stimulated mobility and fusion data were characterized by the sum of two zero-truncated Gaussian cumulative distributions.

To better visualize the cellular response distributions, we present the pooled data as “bee swarm” plots (Fig. 4 A-B) that clearly illustrate the bimodal nature of the data. We observed two distinct populations for both the insulin-stimulated mobility and fusion rate data, with one of these populations coinciding with the basal state; we refer to this latter subpopulation as “insulin-refractory” (Fig. 4A-B).

Based on these findings, we propose that the observed insulin response distributions are best modeled by a bimodal population comprising two states: insulin-refractory and insulin-responsive adipose cells. While the insulin-refractory state of adipose cells may not be identical to the basal state with respect to all cellular processes, it is statistically indistinguishable from the basal condition with respect to GSV trafficking and fusion.

**Fig 5:**
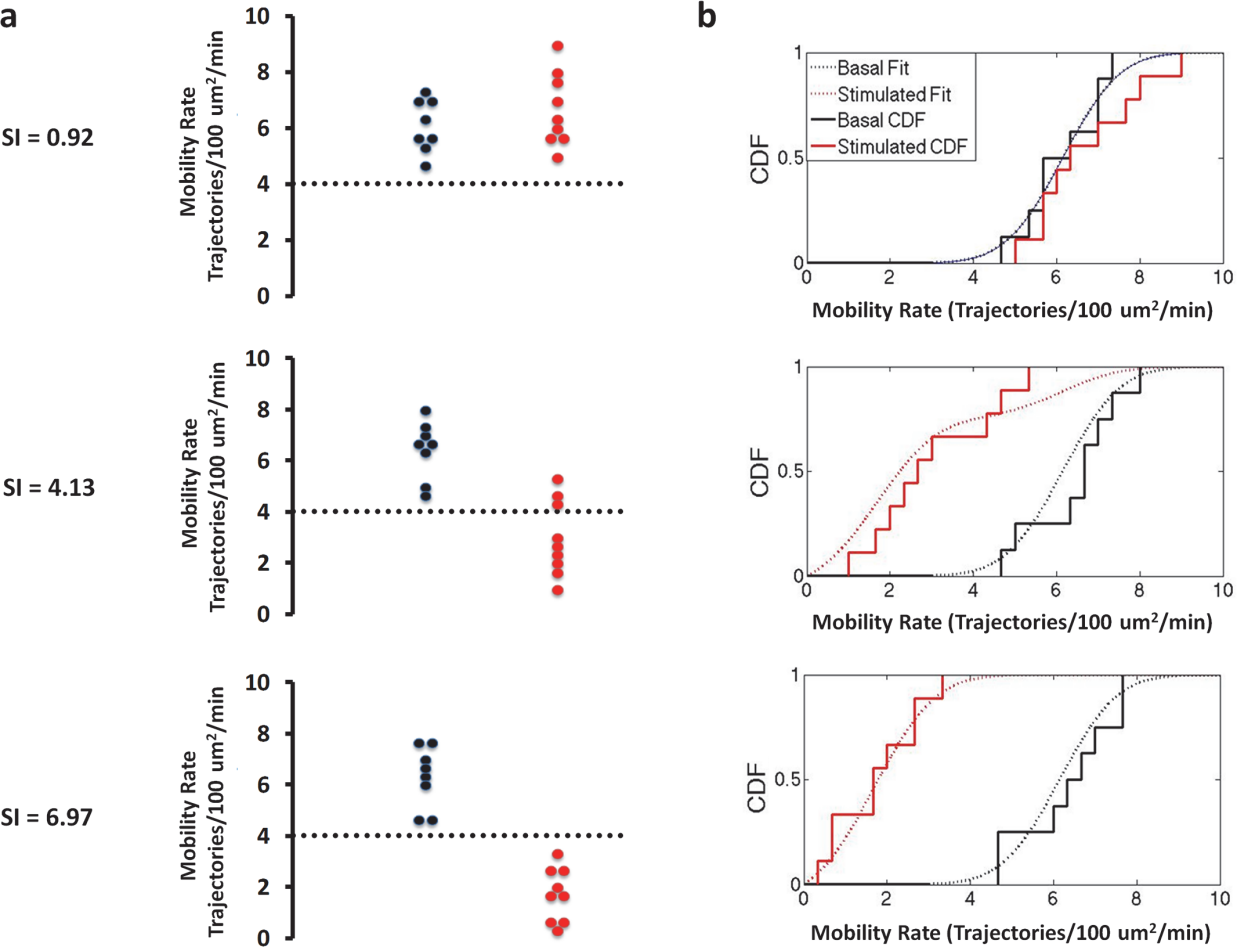
Representative “Bee swarm” plots (a) and empirical cumulative distribution functions (b) for basal (black) and insulin-treated (red) GSV mobility rates measured in adipose cells isolated from subjects with varying degrees of insulin sensitivity reveals two populations at the individual subject level. Dashed lines represent cluster boundaries calculated using the K-means clustering function. At low SI both the basal and insulin stimulated distributions are fit by a single, basal function. With increasing SI, increasing number of cells shift from the basal into the insulin-stimulated state characterized by low GSV mobility (less than 4 Trajectories/100 μm^2^/min).

**Fig 6:**
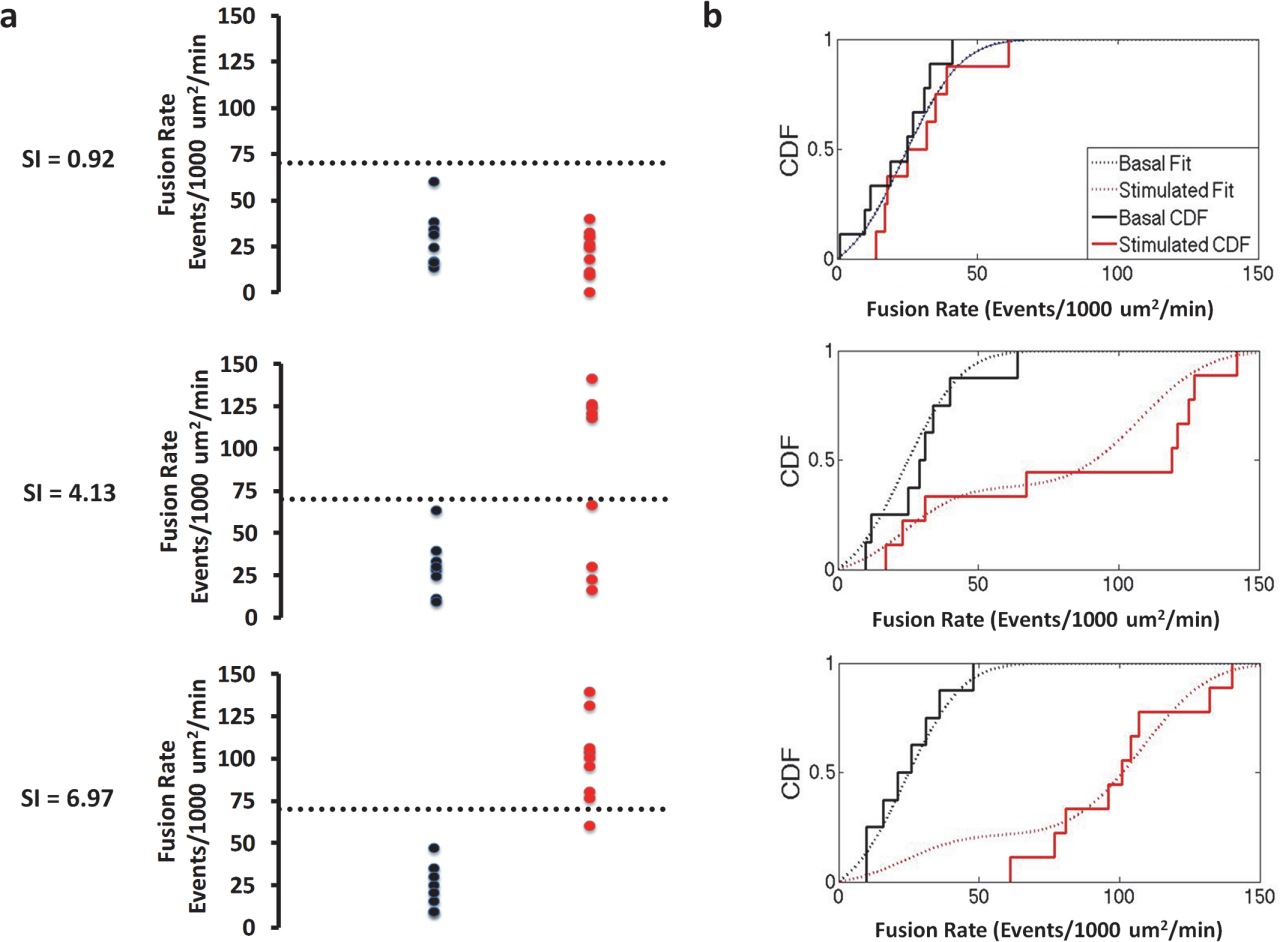
Representative “Bee swarm” plots (a) and empirical cumulative distribution functions (b) for basal (black) and insulin-treated (red) GSV fusion rates measured in adipose cells isolated from subjects with varying degrees of insulin sensitivity reveals two populations at the individual subject level. Dashed lines represent cluster boundaries calculated using the K-means clustering function. At low SI both the basal and insulin stimulated distributions are fit by a single, basal function. With increasing SI, increasing number of cells shift from the basal into the insulin-stimulated state characterized by high fusion rate (greater than 70 Events/1000 μm^2^/min).

To perform a quantitative analysis of the basal and insulin-stimulated distributions in GSV mobility and fusion rates, the pooled adipose cell data were represented as empirical cumulative distribution functions (CDF, Fig. 4 C, D) and fit to model CDFs with one or more Gaussian distributions. The basal distributions were characterized by zero-truncated Gaussian cumulative distributions (Fig. 4 C,D, black dotted lines), while the insulin-stimulated distributions were best characterized by two zero-truncated Gaussian cumulative distributions, one of which matched the basal distribution parameters (Fig. 4 C,D red dotted lines). If our hypothesis is correct, we would predict that the adipose cell data from individual subjects should be described by one (basal) or two (basal plus insulin-stimulated) Gaussian cumulative distributions. However, the distribution models comprise too many parameters to accurately describe the individual data sets. To resolve this problem, the means and standard deviations from the pooled data fits were used as fixed parameters, reducing our model to only one free parameter, the fraction of adipose cells, in each data set, present in the basal state. We next fit the pooled cellular data from all 19 subjects to determine the fraction of refractory adipose cells and verified if these models satisfactorily describe the data over the entire SI range. Scatter plots and empirical CDFs, and their model fits are presented in Figs. 5 and 6 for three representative subjects with low, medium, and high SI (for Mobility (Fig. 5), fractions (95% confidence) = 1 (.15), 0.24 (.13), 0 (.22), R^2 = 0.91, 0.87, and 0.88, respectively and for Fusion (Fig. 6), fractions (95% confidence) = 1 (.21), 0.38 (.21), 0.21 (.13), R^2^ = 0.82, 0.85, and 0.86, respectively). With increasing SI, increasing insulin-stimulated distribution fractions are observed; however, consistent with previous observations, even in the most insulin-sensitive subjects some adipose cells are observed to exhibit basal-like GSV mobility and fusion rates (Fig. 1).

Thus, GLUT4 translocation in individual adipose cells isolated from human subjects is better characterized as an all-or-none (bimodal) response than a graded response. Our single cell analysis of human adipose cells reveals a bimodal distribution for each of two characteristic properties of GSV, mobility and fusion, affected by insulin. This invariance in the underlying distributions, refractory and insulin-stimulated, with a clearly determinable fraction of cells populating these two states in each subject, is consistent with a threshold or switch-like transformation from the basal to the stimulated state upon addition of insulin. Support for such a response to insulin, termed ‘hard-wired’ at the level of single cells in differentiated 3T3-L1 adipocytes [12], shows that the unique GLUT4 trafficking responses exhibited by individual primary adipose cells are highly reproducible, not merely a stochastic outcome of a multi-stable process.

**Fig 7:**
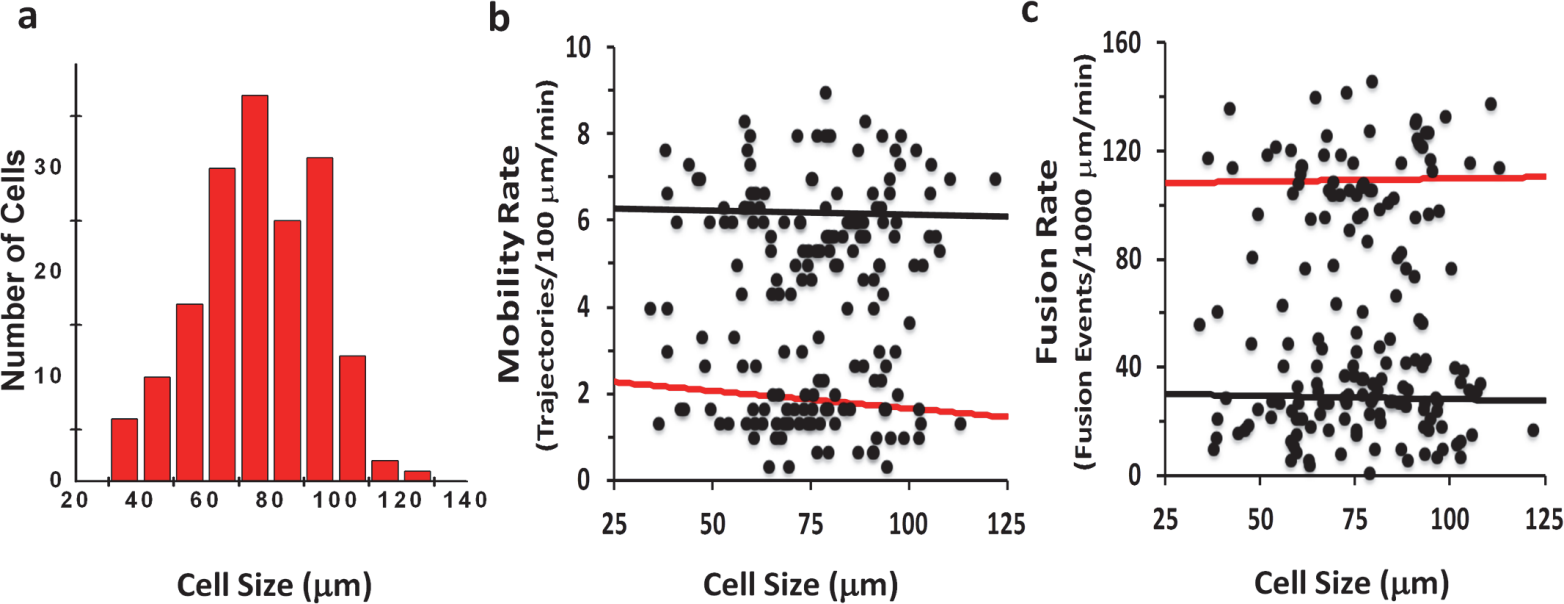
GSV mobility and Fusion rates do not correlate with adipocyte size. Histogram of estimated cell sizes obtained from TIRF projection (a). Mobility and fusion rates as a function of estimated cell sizes (b and c). The slopes of the linear fits for both mobility and fusion in the basal (black) and insulin stimulated (red) are not significantly different from zero indicating there is no correlation with adipocyte size.

To determine a mechanism giving rise to the cellular bimodal response described here, we first thought of the well-documented heterogeneity in adipose cell size [13, 14]. However, while we did not systematically study adipose cells of widely different size, we rule out this possibility because among the cells we did study in our experiments, cell size distribution was unimodal (Fig. 7), with no correlation observed between the measured GSV trafficking and fusion parameters, and the estimated cell size (Fig. 5 B,C). We cannot at this time judge other parameters of cellular heterogeneity, such as cell. age and/or the recently described potential for trans-differentiation from white to beige adipose cells [15]. More likely, in addition to the genetic factors that influence cell physiology, adipose cells *in situ* are exposed to multiple extracellular factors that bind to cell surface receptors, such as hormones and cytokines [16, 17]. Metabolites that enter cells directly, such as glucose and fatty acids, may also have a role [4, 18]. The finding that glucose entry regulates organelle trafficking by the covalent attachment of a glucose moiety to an intracellular protein regulator of the specific microtubule motor activity in *Drosophila*, where insulin also functions to stimulate GLUT4 trafficking [19], demonstrates how O-GlcNAc transferase could operate in adipose cells to provide a primary defect [20]. Furthermore, heterogeneity may occur as a consequence of interactions between the immune system and individual adipose cells; immune cells present in adipose tissue have been shown to play dual functions as they are capable of both inducing local insulin resistance and increasing insulin-sensitivity of adipose cells [21, 22]. Nevertheless, whatever the factor(s) may be, adipose cells in culture retain a “memory” of the physiological state of the subject from which they are removed, consistent with the concept that the observed cellular characteristics may be a driving force in systemic physiology rather than a consequence.

In general, bimodal or switch-like responses to a signal indicate that the signaling system possesses both negative feedback and feed forward loops that, depending on parameters inherent to an individual cell, determine its insulin-responsive state [23, 24]. We envision multiple inputs to the adipose cell, together with genetic background [4], summing to an all-or-none decision for a common final pathway through which adipose cells signal to the body, either by altered metabolic function leading to a redistribution of metabolic fuels or by altered secretion of as yet unidentified and unconfirmed adipokines, or both, in either case impacting on other organ systems [1, 14, 16, 17]. On the other hand, we have not ruled out the possibility that individual signaling pathways may themselves respond in a bimodal fashion.

### The refractory fraction determines the population response

**Fig 8:**
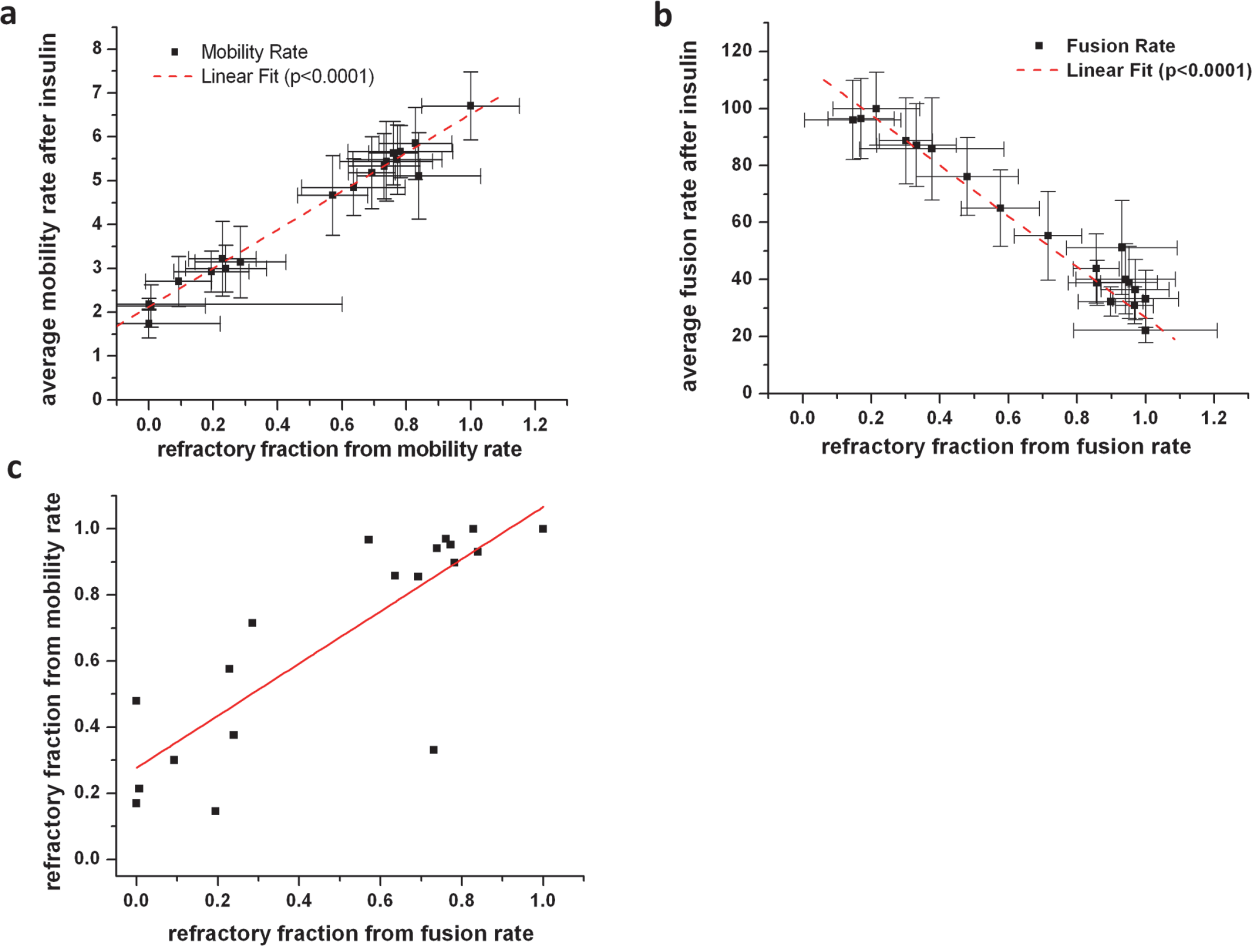
The average insulin response of the population of adipose cells (measured per subject) is determined by the change in the fraction of refractory cells, defined as the basal-like fraction present in the insulin stimulated state and derived from distributional fitting of individual subject insulin responses. The average values for both mobility (a) and fusion (b) rates measured for populations of cells was linearly correlated with the fraction of refractory cells. (c) The correlation between refractory fractions determined from mobility and fusion data (R^2^ = 0.67) does not pass through the origin.

Interestingly, the average response of the population of adipose cells (measured per subject) is almost exclusively determined by the change in the fraction of refractory and responding cells. The average values for both mobility and fusion rates measured for populations of adipose cells have a linear dependence on the fraction of refractory cells (Fig. 8 A, B). Linear correlations between the average cell response and the fraction of refractory cells across the whole range of subjects with SI from 0.16 to 11 indicate that the average response is determined primarily by the change in the fraction of refractory cells rather than by a change in the amplitude of the response of non-refractory cells. Indeed, if the number of refractory adipose cells increases, it is expected to result in a lower average response for a cell population. However, this is only true if the underlying distribution parameters of the cellular responses (i.e. characteristic peak values for refractory and responding cells) do not change significantly with SI, which is exactly the case with GSV mobility and fusion rates.

Further, the correlation plot between refractory fractions determined from mobility and fusion data, although linearly correlated at R^2^ = 0.67, do not exhibit a 1:1 correlation (Fig. 8C). While it is expected for the insulin-stimulated increase in fusion rate to be dependent on the preceding decrease in GSV mobility, the observed deviation from this dependence is probably due to the existence of independent factors that affect insulin regulation of the GSV mobility and fusion processes separately. Interestingly, the highest variance (deviation from the correlation line) between refractory fractions determined from mobility and fusion data occurs in the middle range of values (0.2–0.8) characteristic for subjects with intermediate values of SI (see also Fig. 1).

### The refractory fraction correlates with systemic parameters

**Fig 9:**
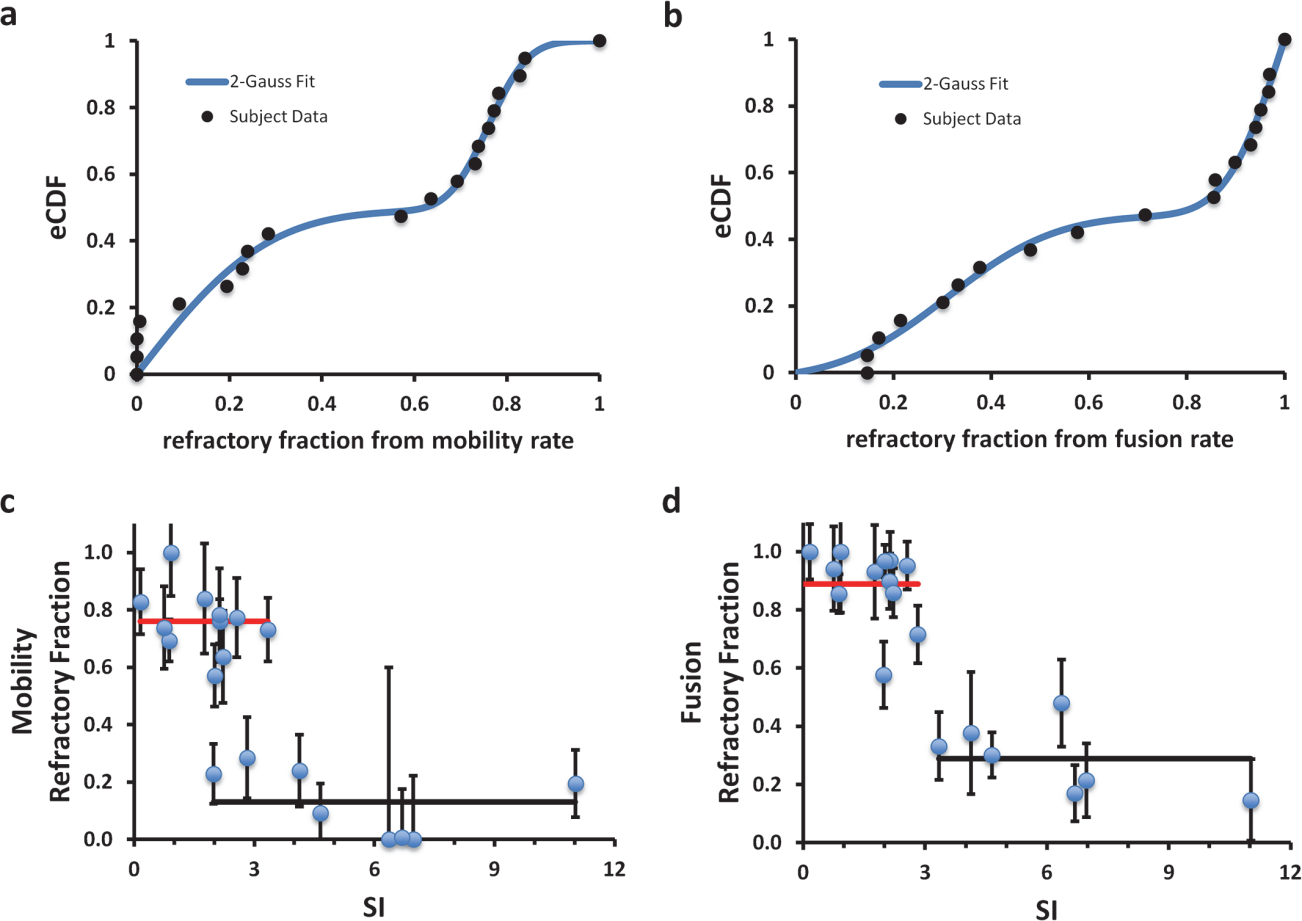
Refractory fractions obtained from both mobility (a) and fusion (b) data, cluster into two populations whose CDF is described best by the sum of two 0–1 truncated gaussians (blue fit). The refractory fractions correlate with decreasing insulin SI of the donor subject. The solid lines (high, red and low, black) are the cluster population averages drawn to span the range of data. The overlap region along the SI axis is where switching behavior is expected to occur since subjects can have similar SI values but different refractory fractions. Please note that the error bars reflect the failure fraction fitting error (95% confidence) and not the population variance in the experimental data.

Since adipose cells may actually contribute directly to systemic insulin resistance [1, 4, 8], we next investigated if the fraction of refractory cells in the insulin-stimulated state increase with increasing values for the insulin resistance seen in our subject population. The fractions of refractory adipose cells were determined from the CDF fits (Fig. 5, 6) and plotted for each subject (Fig. 9, n = 19). Whereas the subject SI values are best described by a unimodal distribution, the insulin-stimulated refractory fractions for both fusion and mobility are best described by bimodal distributions (a mixture model of two truncated Gaussian distributions) (Fig. 9 A, B). The mean value of each of the two characteristic refractory fractions observed for both fusion and mobility in the insulin-stimulated state was used to model the SI dependence (Fig. 9 C, D). Indeed, weighted sum of squares error (SSE) analysis confirms that this two-component model better describes the data than a single correlation line (for fusion and mobility, the SSE for the linear fits are 0.025 and 0.044, respectively, compared to 0.010 and 0.009 for the variation around the means of the two characteristic refractory fractions symbolized by the red and black lines), although the latter are also statistically significant.

**Fig 10:**
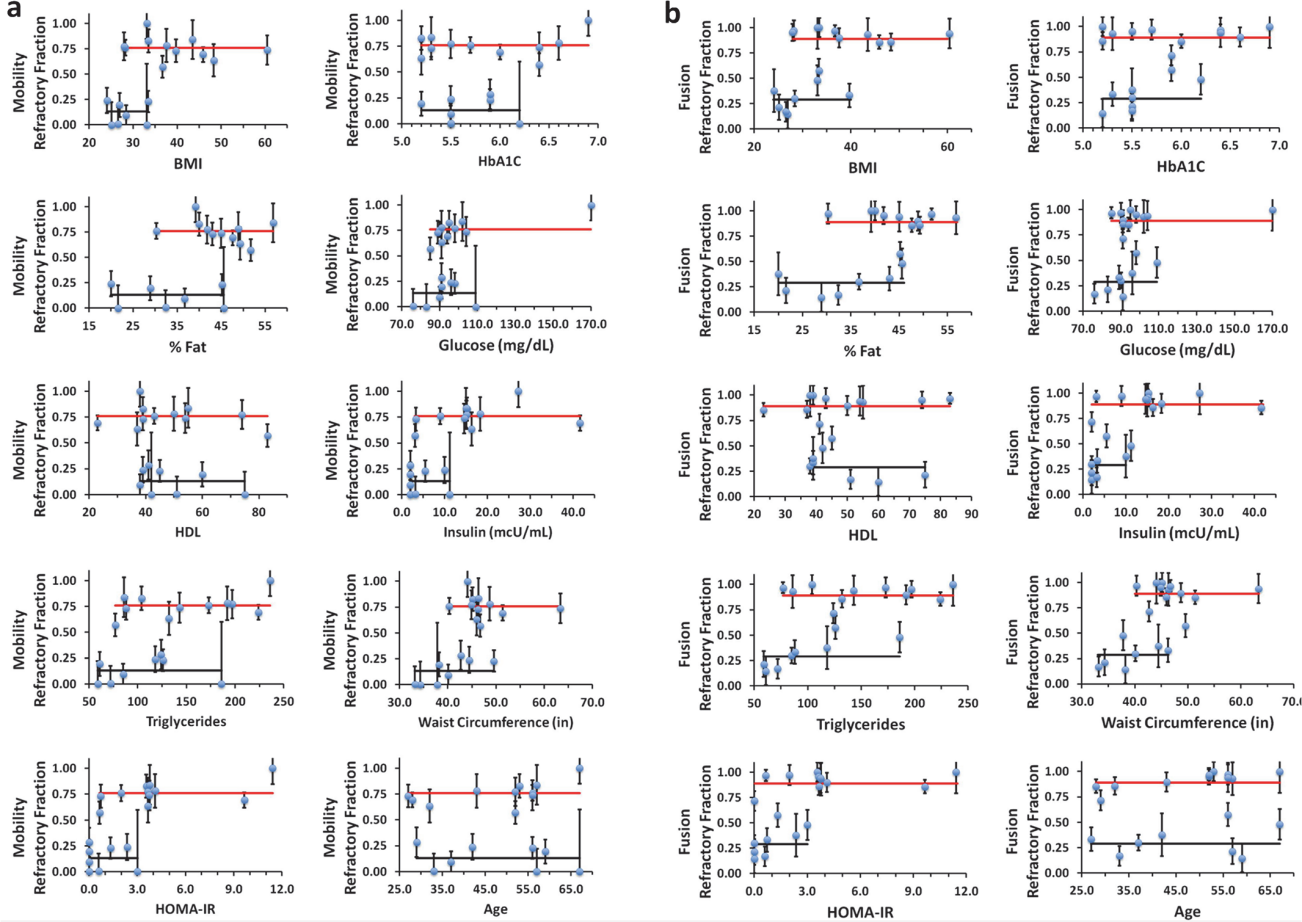
The refractory fractions obtained from analysis of mobility (a) and fusion (b) data plotted as a function of clinical parameters of the donor subject such as BMI, %Fat, HDL, triglycerides, HOMA-IR, waist circumference, age, HbA1C, glucose, and insulin. Refractory fractions cluster into two populations: high (red) and low (black). The solid lines are the cluster averages drawn to span the range of data. The overlap region along the clinical axis is variable with clinical values: e.g. for age the overlap region spans the entire range, while for parameters such as glucose, insulin, BMI and HOMA-IR the overlap region is narrower.

In addition to their relationship with SI, we plotted the refractory fractions against the other clinical measures of insulin resistance and associated risk factors (Fig. 10). Unexpectedly, the subjects themselves comprise two populations in which their isolated adipose cells *in vitro* in the insulin-stimulated state exhibit either a high or low fraction of refractory cells. We also note a similar two-population relationship when we analyzed the average values of the isolated adipose cell GSV mobility and fusion values per subject as previously reported For many of the clinical parameters, the segregation of subjects by the level of insulin-refractive adipose cell fraction reveals two domains with variable transition or overlapping regions, where the percentage of subjects varies with the clinical parameter measured (Fig. 10). While some clinical parameters, i.e. age, are equally represented at both insulin-refractive adipose cell levels, others show clear transitions; depending on the values one accepts as indicative of clinical risk for the metabolic syndrome [25, 26], these transitions appear to be somehow related to these risk factors, i.e. HbA1c (>6.2), HOMA-IR (>3), serum glucose (>110 mg/dL), fasting insulin (>10 mcU/ml), BMI (>35), and %Fat (>45). All subjects whose insulinemia, serum glucose, HbA1c, BMI, and %Fat are above these thresholds, have most of their adipose cells in the insulin-refractory state. However, the inverse is less clear: some subjects who have significant fractions of refractory adipose cells may still have many of these clinical indices in the normal range. This could either be due to genetic differences between the two populations, or environmental effects such as diet, or both. We have not considered in detail here the indirect nature of the clinical parameters used or their limitations as they are typically assessed in the clinical environment. Nevertheless, examination of the GSV responses to insulin in isolated adipose cells per subject *in vitro* appears to allow division of this subject population into two general groups, one of which may be at risk of developing severe insulin resistance and type 2 diabetes.

Recent studies indicate genetic and other reasons for protection for some individuals from risk factors for insulin resistance and diabetes [26–28]. Perhaps specific genetic factors contributing risk for, or protection from, systemic insulin resistance will be best studied in the narrowest transition zone of clinical indices (the overlap region where refractory fraction can be both low and high, Fig. 9 C, D and Fig. 10) as these subjects will be matched in many of the contributing factors yet still show divergent *in vitro* cellular indices.

On the other hand, the tendency for adipose cells to be either refractory or responsive in a specific subject clearly reflects a physiological state essential to systemic homeostasis. Indeed, the refractory cells may be unlike basal cells. They may instead be responding to insulin via pathways that do not lead to GSV exocytosis, like inhibition of lipolysis and adipokine secretion. In general, more basic science research is needed to study the transition between the physiological aspects of insulin resistance that are selectively advantageous for optimal regulation of metabolic fuel distribution, and pathological aspects of insulin resistance that are destructive. Ultimately, understanding the mechanism of this newly discovered switching process and the determinants of the varying fractional responses to insulin are fundamental to developing interventions that will control the detrimental effects of systemic insulin resistance. If the adipose cells in the subcutaneous fat depot are “hard wired” as implied by their retention of a close relationship *in vitro*, in the presence of constant culture conditions and milieu, to donor subject systemic homeostasis *in vivo*, we suggest that the physiological switching mechanism at the adipose cellular level may ultimately drive systemic SI via altered adipose cellular functions such as fuel metabolism and adipokine secretion.

## Methods

### Subjects

Biopsies of subcutaneous abdominal fat were obtained from human subjects with varying degrees of obesity, enrolled in the study “Physical and Behavioral Traits of Overweight and Obese Adults” registered at clinicalTrials.gov (NCT00428987). This study was approved by the institutional review board of National Institute of Diabetes and Digestive and Kidney Diseases. All patients provided written consent to participate in this study. The consent was obtained by qualified associate investigators prior to the subject’s participation and placed in the medical record and research file. Insulin sensitivity index (SI) was calculated from glucose and insulin measurements using the MinMod Millennium (6.02). Body fat percent was determined by dual energy x-ray absorptiometry (DXA) using a total body scanner (Lunar iDXA, GE Healthcare, Madison, WI).

### Reagents

IRAP-pHluorin was kindly provided by T. Xu and the IRAP-GFP, by J.E. Pessin. Construction of HA-GLUT4-mCherry has been described previously [29]. Carrier DNA was from Boehringer (Mannheim). BSA (fraction V) and collagenase were obtained from Intergen (Purchase, NY) and Worthington (Lakewood, NJ), respectively. DMEM, and human recombinant insulin, were obtained from Invitrogen (Grand Island, NY). Insulin was prepared as a stock (250 IU/ml) and diluted into KRBH buffer, 1% BSA, to a final working concentration of 0.1 IU/ml.

### Adipose Cell Isolation and Transfection

Adipocytes were isolated from tissue biopsies by collagenase (1 mg/ml) digestion (40–60min at 37C). Following isolation, adipocytes were washed twice with 25ml KRBH buffer containing 1% BSA (fraction V) and resuspended into DMEM containing 25 mM glucose, 25 mM HEPES, 4 mM L-glutamine, 200 nM(−)-N 6-(2-phenylisopropyl)-adenosine, and 50 IU/ml penicillin, 50 μg/ml streptomycin, to a cytocrit of 40%. Electroporation was carried out in 0.4-cm gap-width cuvettes (Bio-Rad) using a T810 square wave pulse generator (BTX) with one pulse of 6ms at 400V. All plasmids were used at the final concentration of 8 μg/ml, together with 100 μg/ml of carrier DNA. Tranfected cells were washed once in DMEM, and incubated overnight at 37 °C, 5% CO_2_ in DMEM containing 3.5% BSA.

### Microscopy and Image Analysis

Cells were imaged using an objective-based TIRF setup designed around a Nikon TI microscope and coupled with a custom-built laser combiner system equipped with 405/488/561 nm lasers (Coherent)[30]. Using the adipose cell preparation from each subject, we performed 3–4 sequential microscopy studies. For each individual study, an aliquot (5 μl) of the isolated adipose cells were transferred from the cell suspension (kept in a 15-ml tube in an incubator) into a pre-heated microscopy chamber filled with KRBH buffer (1% BSA, pH 7.4), and maintained at 37°C using a temperature controller (Delta-T, Bioptechs). Three to four (3–4) transfected cells in basal medium were located, digitally tagged, and sequentially recorded by TIRF (2 min at 1fps). After application of 0.1 IU/ml insulin to the microscopy chamber, the same 3–4 cells were located by the automated microscope stage (MS-2000, ASI) using the tagged X-Y coordinates stored in the Micro-Manager position list, and TIRF recordings were repeated for each cell (2 min at 1fps) in the specified time window (15–20 min after addition of insulin). The overall time the cells spent in the microscopy chamber did not exceed 60 min per study. The level of the response of individual cells did not show any correlation with either the order they were imaged (e.g. time window: 15–16, 17–18, and 19–20 min after insulin) or the sequential number of the experiment (the time between the 1st and 3rd experiments was about 3 h, which corresponds to 24–27 h after transfection). Trafficking and fusion of GSV labeled with GLUT4-mCherry/Irap-pHluorin was analyzed as described previously [6, 9]. GSV Mobility rate was estimated as the number of mobile vesicles (with trajectory length >2 μm) detected within a 10x10 μm region of interest (ROI) during one minute of recording. Fusion rate was calculated as the number of transient spikes of IRAP-pHluorin fluorescence per unit area and renormalized to 1000 μm^2^.

### Statistical Analysis

All cell response data were pooled and the normalized cumulative distribution functions (nCDF) were determined for mobility and fusion rates. The nCDF was fit using a sum of two characteristic cumulative distributions. Both mobility and fusion rate data sets were well described using a five parameter fit (μ_1_, σ_2_, μ_2_, σ_2_, w) where μ and σ are the mean and standard deviations of a zero-truncated gaussian distribution and w is the fraction of the first gaussian distribution and (1-w) is the fraction of the second. The nCDF were then calculated for cells from each individual subject and fit using a one parameter sum of these two characteristic distributions where the means and standard deviations from the “pooled data” fit were fixed; only the fraction of refractory cells (w) was allowed to vary The uncertainties in refractory fraction values are represented by the 95% confidence values obtained from the one parameter fits. Since the fractions span the range 0–1, 0:1 truncated gaussians were used to fit the refractory fractions to obtain the two characteristic mean values represented by the red and black lines in Figs. 9C,D and 10.
